# Rozwój w Pierwszym Roku życia Noworodków Urodzonych Przedwcześnie – Doniesienie Wstępne

**DOI:** 10.34763/devperiodmed.20182203.247254

**Published:** 2018-10-04

**Authors:** Agnieszka M. Zdzienicka-Chyła, Krystyna Mitosek-Szewczyk

**Affiliations:** 1 Klinika Neurologii Uniwersytetu Medycznego w Lublinie, Polska

**Keywords:** wcześniaki, monachijska funkcjonalna diagnostyka rozwojowa, fizjoterapia, premature babies, Munich Functional Developmental Diagnostics, physiotherapy

## Abstract

**Wstęp:**

Noworodki urodzone przed czasem stanowią blisko 7% wszystkich urodzonych dzieci i jednocześnie przybywa dzieci urodzonych jako ekstremalnie małe wcześniaki. W Parlamencie Europejskim już w roku 2011 priorytetowym celem zdrowotnym oznaczono problem wcześniactwa. Podkreśla się, że noworodki urodzone przedwcześnie wymagają długotrwałej opieki specjalistycznej.

**Cel pracy:**

Celem pracy była wstępna analiza poziomu rozwoju noworodków urodzonych przed 37. tygodniem życia płodowego z uwzględnieniem różnych obszarów rozwoju, oraz poszukiwanie różnic w poziomach rozwoju noworodków urodzonych przedwcześnie i o czasie.

**Materiał i metody:**

Badaniem objęto dwie grupy dzieci: I grupa **–** dzieci urodzone przedwcześnie (pomiędzy 25. a 36. tygodniem życia płodowego) badane w wieku urodzeniowym 11,5-12,5 miesiąca, II grupa – dzieci urodzone o czasie oceniane w wieku 11,5-12,5 miesiąca. Ocenę przeprowadzono z wykorzystaniem Monachijskiej Funkcjonalnej Diagnostyki Rozwojowej. Analizie poddano 50 kwestionariuszy noworodków urodzonych przed czasem oraz 30 dzieci urodzonych o czasie. Oceniano motorykę dużą, motorykę małą, samodzielność i percepcję.

**Wyniki:**

W ocenie ogólnej 80% dzieci z grupy I podejmowało aktywność zgodną z wiekiem urodzeniowym, jednak uwzględniając analizę rozwoju w poszczególnych sferach 50% dzieci z grupy I podejmo-wało aktywność zgodną z wiekiem urodzeniowym. Analizując wartości średnie, odnotowano istnienie relacji między poziomem wcześniactwa, a poziomem aktywności prezentowanej przez badane dzieci. Im większy stopień wcześniactwa tym bardziej maleje poziom aktywności prezentowanej przez dzieci. Największe trudności w grupie I odnotowano u dzieci w zakresie percepcji i samodzielności.

**Wnioski:**

Poziom aktywności prezentowany przez dzieci urodzone przedwcześnie w wieku 12 miesięcy od urodzenia jest zależny od poziomu wcześniactwa. Noworodki urodzone przedwcześnie wymagają diagnostyki w poszczególnych różnych sferach rozwoju.

## Abstract (de)

**Introduction:**

Premature babies account for almost 7% of all newborns and the number of extremely premature infants is increasing. In the European Parliament the problem of premature birth was identified as a priority health task as early as 2011. It is emphasized that the problems of a premature newborn do not end with discharge from hospital.

**Objective:**

The aim of the paper was a tentative analysis of the development level of infants born before the 37^th^ week of intrauterine life, taking into account various areas of development and searching for differences in the levels of the development of newborns born prematurely and on time.

**Material and methods:**

The research covered two groups of babies: group I – infants born prematurely (between the 25^th^ and 36^th^ week of their intrauterine life) examined in their 11.5-12.5 month, group II – infants born in due time, examined in their 11.5-12.5 month. Evaluation was carried out in accordance with the Munich Functional Developmental Diagnostics. The analysis comprised 50 questionnaires of prematurely born babies and 30 infants born in due time. Gross and fine motor skills were assessed, as well as autonomy and perception.

**Results:**

In our general evaluation, 80% of the children from group I were active in accordance with their birth age. However, as regards the analysis of development in particular areas, 50% of the children from group I undertook activity compliant with their birth age. The analysis of average values indicates the relationship between the level of prematurity and the level of activity presented by the children examined. As the level of prematurity increases, the level of activity presented by the children decreases. The greatest difficulties in group I were noted with regards to perception and autonomy.

**Conclusions:**

The level of activity presented by the children born prematurely at the age of 12 months depends on their level of prematurity. Newborn babies who were born prematurely require diagnostics specifying diffierent spheres of development.

## Wstęp

Noworodek urodzony przedwcześnie to dziecko urodzone przed 37. tygodniem ciąży. Ze względu na wiek przyjścia na świat wyróżnia się:

– średnie wcześniaki – urodzone między 32. a 36. tygodniem ciąży,
– skrajne wcześniaki – urodzone między 28. a 31. tygodniem ciąży,
– ekstremalnie skrajne wcześniaki – urodzone przed 27. tygodniem ciąży. [1]

W procesie diagnostycznym rozwoju noworodków urodzonych przedwcześnie wskazane jest odnoszenie oceny rozwoju do wieku skorygowanego, a nie do wieku urodzeniowego, aż do momentu ukończenia przez dziecko co najmniej 18 miesięcy. Przy czym rozwój dziecka powinien bezwzględnie podlegać monitorowaniu do 2. roku życia wieku skorygowanego [2]. Zgodnie z wytycznymi koncepcji neurorozwojowej NDT Bobath, korekcję wieku urodzeniowego zaleca się stosować do drugiego roku życia dziecka. W 2. roku życia nie stosuje się już pojęcia wieku korygowanego i rozwój dziecka odnosi się do wieku urodzeniowego, jednak opinie w tej kwestii są niejednoznaczne [3]. Uważa się też, że u części wcze-śniaków wiek może być korygowany nawet do 3. roku życia [4]. Brakuje więc jednoznacznych wytycznych, jak długo oceniać dziecko według wieku korygowanego. Nie ma także konkretnych wytycznych na temat tego czy dzieci urodzone jako skrajne wcześniaki lub ekstremalnie skrajne wcześniaki dłużej oceniamy według wieku korygowanego, dając im czas przed włączeniem interwencji. Na uwagę zasługuje inna sytuacja dziecka urodzonego przed czasem, zgodnie ze stanowiskiem Polskiego Towarzystwa Ginekologicznego „zdrowie kształtuje się od wczesnego okresu życia osobniczego, tak więc od prawidłowego przebiegu ciąży i porodu jest zależny fizyczny oraz intelektualny rozwój dziecka” [5].

Noworodek urodzony przedwcześnie jest pacjentem wyjątkowo wymagającym w zakresie opieki i prowadzonej pielęgnacji z uwagi na niedojrzałość poszczególnych narządów. Przeżywalność w tej grupie dzieci jest zależna od urodzeniowej masy ciała dla dzieci ważących 750-1000 gramów wynosi obecnie ponad 90%, z masą od 500g do 750 g 50% do 75%, a najmniejszych ważących do 500g tylko 1-5%. Jednocześnie z uwagi na niedojrzałość zmagają się one z wieloma problemami i zwiększonym ryzykiem wystąpienia nieprawidłowości. Do zagrażających zaburzeń klinicznych noworodków urodzonych przedwcześnie należą między innymi niewydolność oddechowa, posocznica, martwicze zapalenie jelit czy zapalenie płuc [6, 7].

## Cel pracy

Celem pracy była ocena rozwoju noworodków urodzonych przedwcześnie, w 12 miesiącu życia z uwzględnieniem różnych sfer rozwojowych. Celem badań było uzyskanie odpowiedzi na pytanie jaki procent noworodków urodzonych przed czasem osiąga umiejętności zgodne w wiekiem urodzeniowym w pierwszym roku życia. Jednocześnie w pracy poszukiwano różnic występujących pomiędzy poziomem rozwoju noworodków urodzonych przedwcześnie, a dzieci urodzonych o czasie.

## Materiał i metody

Badaniem objęto II grupy dzieci: I grupa − noworodki urodzone przedwcześnie oceniane w wieku 11,5-12,5 miesięcy wieku urodzeniowego, II grupa − dzieci urodzone o czasie oceniane w wieku 11,5-12,5 miesiąca. Przeprowadzono ocenę zgodnie z Monachijską Funkcjonalną Diagnostyką Rozwojową (MFDR) z wyszczególnieniem sfer rozwoju: diagnostyka wieku chodzenia, sprawności manualnej, percepcji oraz samodzielności. W badaniu uwzględniono także ocenę ogólną czyli średnią osiąganych rezultatów w badaniu poszczególnych sfer.

Diagnostyka wieku chodzenia (ruchu ciała) obejmowała ocenę umiejętności z zakresu motoryki dużej (9 aktywności), między innymi: przesuwanie się kilka kroków wzdłuż mebli, chód z przytrzymaniem za obie ręce i utrzymaniem ciężaru ciała, utrzymywanie samodzielnie pozycji stojącej, chód samodzielny. Diagnostyka wieku sprawności manualnej (7 aktywności) obejmowała aktywności z zakresu motoryki ręki, motoryki precyzyjnej, między innymi: uderzanie poziomo klockami o siebie, chwyt zgiętym palcem i palcem wskazującym, przesuwanie samochodzikiem. Diagnostyka percepcji (5 aktywności) dotyczyła pojmowania zależności i postrzegania zmysłowego, oceniano między innymi czy dziecko znajduje przedmiot schowany pod kubkiem, podąża wzrokiem za palcem wskazującym określony kierunek, przyciąga do siebie zabawkę pociągając za sznurek. Diagnostyka samodzielności (5 aktywności), które oceniano na podstawie obserwacji, jak i wywiadu z rodzicami, obejmowało między innymi, samodzielne zdjęcie czapki z głowy, wkładanie do ust pokrojonego na kawałki chleba, picie z trzymanego przez kogoś kubka (kubeczek z normalnym brzegiem podtrzymywany przez dorosłego).

Badanie uzupełniono analizą dokumentacji medycznej (odnośnie stanu dziecka po urodzeniu, masy urodzeniowej, powikłań, prowadzonej rehabilitacji) oraz kwestionariuszem wywiadu rodzica/opiekuna własnego autorstwa (w celu uzupełnienia informacji o ogólnym stanie dziecka). Celowo w pracy oceniano dzieci zgodnie z wiekem urodzeniowym, tak aby ocenić jaki odsetek dzieci podejmuje aktywność wskazaną dla wieku urodzeniowego w pierwszym roku życia. Dodatkowo, jako grupę kontrolną ocenie poddano 30 dzieci urodzonych o czasie.

Na przeprowadzenie badań uzyskano zgodę Komisji Bioetycznej Uniwersytetu Medycznego w Lublinie. Przed przystąpieniem do badań osoba prowadząca badania ukończyła kurs „Monachijskiej Funkcjonalnej Diagnostyki Rozwojowej − pierwszy, drugi i trzeci rok życia”. Uzyskano także zgodę na wykorzystanie MFDR wydaną przez Towarzystwo Krakowskiego Ośrodka Rehabilitacji Wieku Rozwojowego. Wszyscy rodzice bądź opiekunowie prawni dzieci wyrazili pisemną zgodę na badanie.

Badania były prowadzone w obecności rodzica, dołożono wszelkich starań aby ocena każdego dziecka przebiegała w optymalnym dla niego czasie i w spokojnych warunkach, czasem włączano rodzica dziecka w proces badania. Przeprowadzono ocenę 51 badanych w I grupie (uwzględniono 50 całościowo uzupełnionych kwestionariuszy) i 30 badanych w II grupie. Dzieci objęte badaniem nie miały zaburzeń neurologicznych (także brak nieprawidłowości w USG ośrodkowego układu nerwowego), ani zaburzeń genetycznych. Dzieci nie miały stwierdzonych deficytów w obrębie wzroku lub słuchu i w chwili badania nie miały dodatkowych zaburzeń bądź chorób. Część z dzieci korzystała z wsparcia w postaci fizjoterapii. Wyniki analizowano zgodnie z normą minimalną (zachowania realizowane przez 90% dzieci w określonym wieku).

W celu oceny normalności rozkładu zmiennych zastosowano test Shapiro-Wilka. Ocenę zależności między zmiennymi przedstawionymi w skali nominalnej i porządkowej określano przy zastosowaniu testu Chi [2]. Ocenę istotnych statystycznie różnic między zmiennymi przedstawionymi w skali ilorazowej dla dwóch grup oceniano przy zastosowaniu testu Manna-Whitneya oraz dla wielu grup określano przy zastosowaniu ANOVY rang Kruskala-Wallisa i testu Dunna (test wielokrotnych porównań średnich rang dla wszystkich grup), jako testu post-hoc. Przyjęto 5% błąd wnioskowania i związany z nim poziom istotności wynoszący 0,05 (α=0,05). Analizę statystyczną wykonano przy użyciu programu Statistica v.12.5 (StatSoft, Polska).

## Wyniki

Wstępna całościowa ocena rozwoju (bez podziału na poszczególne sfery rozwoju) wykazała, że dzieci z grupy I prezentują średnio aktywność na poziomie 13,1 (±1,2) miesiąca życia (Tabela I).

**Tabela I:** Średni poziom aktywności dzieci w grupie I i w grupie II. Table I. The average level of activity of children in group I and group II.

Sfera rozwoju *Areas of development*	Grupa I *Group 1*	Odchyl. st. Grupa I *Standard deviation Group I*	Grupa II *Group II*	Odchyl st. Grupa II *Standard deviation Group II*	p
**Ocena ogólna *General evaluation***	13,1	± 1,2miesiąca *± 1,2 month*	13,9	± 0,7 miesiąca *± 0,7 month*	**0,000231**
**Motoryka duża *Gross motor skills***	13,6	± 2,5 miesiąca *± 2,5 month*	14	± 2,2 miesiąca *± 2,2 month*	0,105253
**Motoryka mała *Fine motor skills***	13,4	± 1,2 miesiąca *± 1,2 month*	14	± 0,7 miesiąca *± 0,7 month*	**0,000367**
**Percepcja *Perception***	12,4	± 1,4miesiąca *± 1,4 month*	13,7	± 0,8 miesiąca *±0,8 month*	**0,000026**
**Samodzielność *Autonomy***	13,1	± 1,4miesiąca *± 1,4 month*	14	± 0,7 miesiąca *± 0,7 month*	**0,000574**

Wyniki w zakresie motoryki małej, percepcji, samodzielności pomiędzy dziećmi z grupy I i grupy II, a także w ocenie ogólnej są istotne statystycznie. Uwagę zwraca także szerszy zakres zmienności. Wśród dzieci z grupy I, są one grupą bardziej niejednorodną niż dzieci z grupy II.

W tabeli II przedstawiono ocenę ogólną aktywności badanych dzieci na poziomie wieku urodzeniowego. W grupie I w pierwszym roku życia 80% wszystkich dzieci osiągnęło (według MFDR) poziom rozwoju zgodny z wiekiem urodzeniowym. Jednak w grupie dzieci urodzonych przed 27 tygodniem tylko ok. 45% dzieci osiąga umiejętności na poziomie wieku urodzeniowego. Wykazano istotną statystycznie (p=,00004) zależność pomiędzy wiekiem urodzeniowym, a osiąganiem aktywności zgodnej z wiekiem urodzeniowym w ocenie ogólnej.

**Tabela II:** Dzieci uzyskujące w ocenie ogólnej aktywność na poziomie wieku urodzeniowego, z uwzględnieniem poziomu wcześniactwa. Table II. Children achieving in the general evaluation activity compliant with their birth age, taking into account the level of prematurity.

Dzieci osiągające aktywność zgodną z wiekiem urodzeniowym w ocenie ogólnej *Children achieving in the general evaluation activity compliant with their birth age*.	%	n
**Grupa I razem** ***Group I in total***	80%	40
**Grupa II** ***Group II***	100%	30
**z podziałem z uwagi na poziom wcześniactwa** ***taking into account the level of prematurity***	**%**	**n**
**Grupa I 32-36 hbd** ***Group I 32-36 hbd***	92,59%	25
**Grupa I 28-31 hbd** ***Group I 28-31 hbd***	83,33%	10
**Grupa I 27 hbd i poniżej** ***Group I 27 hbd and earlier***	45,45%	5

Analizując wartości średnie w różnych grupach wiekowych w poszczególnych sferach obserwujemy, że im wcześniej urodzone dziecko tym niższy poziom umiejętności osiąga w danej sferze. Różnice istotne statystycznie pomiędzy poszczególnymi grupami zostały zaznaczone na rycinach 1-4.

Analizując uzyskanie przez dzieci aktywności zgodnej z wiekiem urodzeniowym w poszczególnych grupach wykazano istotne statystycznie różnice w każdym z badanych obszarów. Obserwujemy dużą rozbieżność wyników w zależności od stopnia wcześniactwa. Jednocześnie w zakresie samodzielności i percepcji najmniejszy odsetek dzieci podejmuje aktywność zgodną z wiekiem urodzeniowym, przy czym dotyczy to zarówno dzieci z grupy I i II. Te dwa obszary rozwoju wymagają szczególnej obserwacji, zwłaszcza, że dzieci często oceniane są głównie na podstawie zdolności motorycznych (Wykres 1).

Analizując ocenę poszczególnych sfer rozwoju dzieci z grupy I w pierwszym roku życia 56% badanych dzieci osiągnęło we wszystkich ocenianych sferach (według MFDR) poziom rozwoju zgodny z wiekiem urodzeniowym. Wykazano istotną statystycznie zależność (p=,01760) pomiędzy wiekiem urodzeniowym, a poziomem prezentowanej aktywności (w każdej z ocenianych sfer) całościowo oceniając grupę I, grupę II, a także w grupie dzieci urodzonych pomiędzy 32 a 36 tygodniem ciąży (Tabela III).

**Tabela III:** Dzieci osiągające aktywność wieku urodzeniowego w każdej z ocenianych sfer, z uwzględnieniem poziomu wcześniactwa. Table III. Children achieve activity compliant with their birth age in each of the evaluated areas, taking into account the level of prematurity.

Dzieci osiągające aktywność wieku urodzeniowego w każdej z ocenianych sfer *Children achieving activity compliant with their birth age in each of the evaluated areas*	%	n
**Grupa I razem** ***Group I in total***	56,00%	28
**Grupa II** ***Group II***	80,00%	24
**z uwzględnieniem poziomu wcześniactwa** ***taking into account the level of prematurity***	%	n
**Grupa I 32-36 hbd** ***Group I 32-36 hbd***	66,67%	18
**Grupa I 28-31 hbd** ***Group I 28-31 hbd***	58,33%	7
**Grupa I 27 hbd i poniżej** ***Group I 27hbd and earlier***	27,27%	3

Dzieci z grupy I istotnie statystycznie częściej (p=,00022) niż dzieci z grupy II korzystały z fizjoterapii (Wykres 2), 66% w różnym wymiarze godzin korzystało z tego typu wsparcia. Przy czym w badanej grupie zakres wprowa-dzonej fizjoterapii był zróżnicowany, obejmował od 3 do 20 spotkań terapeutycznych (czas trwania jednego spotkania 30 minut).

**Ryc. 1:**
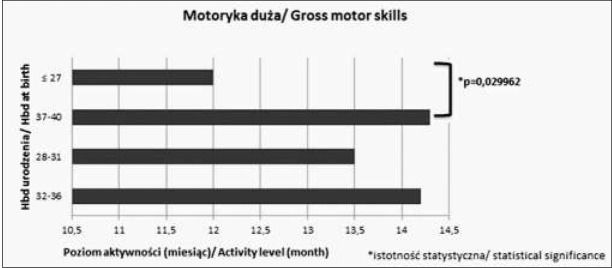
Średnie wartości oceny w poszczególnych sferach rozwoju w zakresie motoryki dużej. Fig. 1. The average values of particular areas of development in the field of gross motor skills.

**Ryc. 2:**
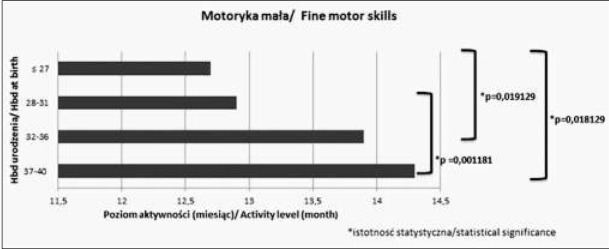
Średnie wartości oceny w poszczególnych sferach rozwoju w zakresie motoryki małej. Fig. 2. The average values of particular areas of development in the field of fine motor skills.

**Ryc. 3:**
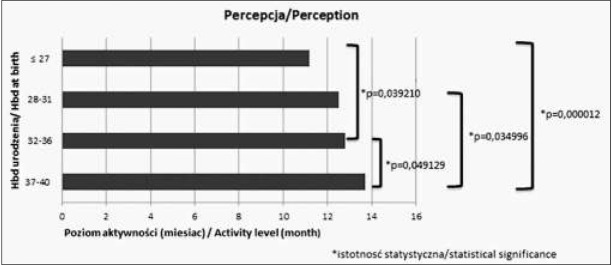
Średnie wartości oceny w poszczególnych sferach rozwoju w zakresie percepcji. Fig. 3. The average values of particular areas of development in the field perception.

## Dyskusja

Zgodnie z przeprowadzonymi badaniami krótszy czas trwania ciąży koreluje ze wzrostem poziomu zaburzeń ośrodkowej koordynacji nerwowej [8]. Badania przeprowadzone we Francji wskazują, że 42% dzieci urodzonych między 24. a 28. tygodniem i 31% dzieci urodzonych między 29. a 32. tygodniem wymagało objęcia specjalistycznym wsparciem, podczas gdy spośród dzieci urodzonych o czasie wsparcia takiego wymagało 16% [9]. Uzyskane w badaniach wyniki wskazujące, że im większy poziom wcześniactwa tym średnio niższy poziom funkcjonowania jest zgodny z dotychczasowymi wynikami badań. W prezentowanych badaniach brakuje jednak informacji o analizie rozwoju dziecka z wyróżnieniem poszczególnych sfer rozwoju. Percepcja i samodzielność to dwa obszary rozwoju dzieci z grupy I, które zwracają szczególną uwagę, ponieważ odnotowujemy tam duże trudności i deficyty. Jednocześnie w literaturze podejmowany jest temat konieczności kontroli ekspozycji dziecka urodzonego przedwcześnie na bodźce, modyfikację cech otoczenia, oraz zwiększonego ryzyka wystąpienia zaburzeń samoregulacji [10, 11].

W literaturze zwraca się także uwagę na trudności rodzicielskie jakie dotyczą rodziców niemowląt urodzonych przedwcześnie – zaburzenie postrzegania dziecka przez rodziców, istnienie stereotypu wcześniactwa, jak również jatrogenne opóźnienie rozwoju dziecka. Rodzic postrzegając własne dziecko jako słabsze i niżej funkcjonujące nie wzmacnia prawidłowo rozwoju dziecka i nie daje mu podstaw do budowania samodzielności [12]. Może to rzutować na późniejsze funkcjonowanie dziecka w tym obszarze.

**Ryc. 4:**
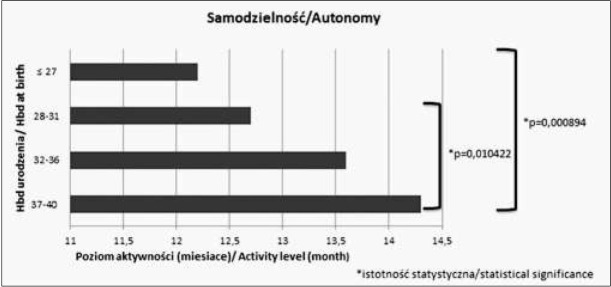
Średnie wartości oceny w poszczególnych sferach rozwoju w zakresie samodzielności. Fig. 4. The average values of particular areas of development in the field of auton

Badania prowadzone w Wojewódzkim Szpitalu Zespolonym w Toruniu wskazały, że z grupy 50 wcześniaków w przeciągu pierwszych dwóch lat życia aż 64% wymagała rehabilitacji, natomiast dzieci urodzone przed 29. tygodniem ciąży wymagają zwykle stałej opieki lekarza oraz rehabilitacji [13]. W grupie dzieci urodzonych przedwcześnie pozycję asymetryczną ciała notuje się u większej liczby badanych niż w grupie dzieci urodzonych o czasie [14]. U dzieci urodzonych przedwcześnie obserwuje się również zaburzenia napięcia mięśniowego [15].

W procesie diagnozy dziecka urodzonego przedwcześnie wiek dziecka powinien być oceniany według wieku skorygowanego do 18. miesiąca życia, część dzieci wyrównuje bowiem deficyty po pierwszym roku życia [16]. W związku z tym zasadne jest przeprowadzenie powtórnej oceny badanych dzieci ponownie w wieku 18 miesięcy i ocena ich funkcjonowania w poszczególnych sferach. Badania przeprowadzone w Trójmieście na grupie 4 i 5 latków wykazały między innymi, że dzieci urodzone przedwcześnie miały obniżony poziom koordynacji błędnikowo-wzrokowej oraz częściej niż ich rówieśnicy urodzeni o czasie prezentowali brak integracji asymetrycznego tonicznego odruchu szyjnego oraz symetrycznego tonicznego odruchu szyjnego [17].

Badania prowadzone w Specjalistycznej Poradni Rehabilitacyjnej dla Dzieci Szpitala Wojewódzkiego w Rzeszowie na grupie 64 dzieci systematycznie poddanych rehabilitacji wykazały, że wczesne wprowadzenie usprawniania wpłynęło na wyrównanie deficytów rozwojowych w ciągu pierwszego półrocza. Jednak podkreśla się często w literaturze, że niemowlęta urodzone przedwcześnie wymagają długotrwałej obserwacji [18]. W literaturze znajdujemy zalecenia wprowadzenia możliwe wczesnej fizjoterapii (przed 6 miesiącem życia dziecka), wskazując, że wczesny moment wsparcia zmniejsza ilość nieprawidłowych doświadczeń sensomotorycznych [19]. W związku z tym analizę rozwoju dziecka przy kolejnych ocenach warto poszerzyć o zakres prowadzonej rehabilitacji, z uwzględnieniem wieku wprowadzonej terapii.

**Wykres 1:**
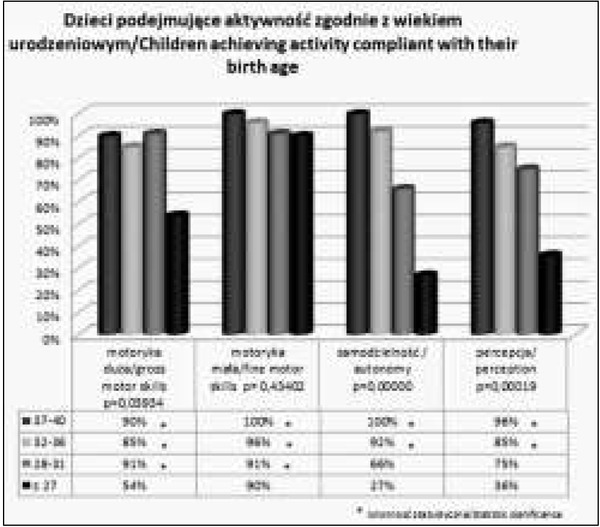
Dzieci podejmujące aktywność zgodnie z wiekiem urodzeniowym (we wszystkich ocenianych sferach), z uwzględnieniem poziomu wcześniactwa. Graph 1. Children achieving activity compliant with their birth age (in all evaluated areas), taking into account the level of prematurity.

**Wykres 2:**
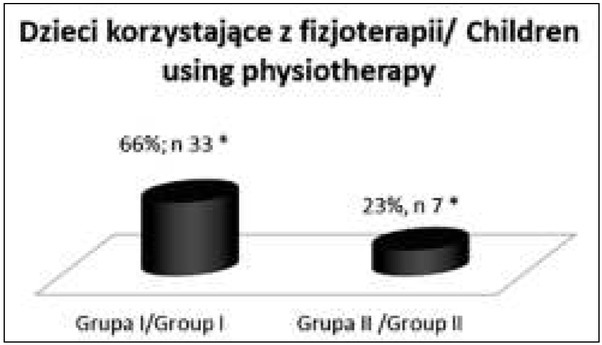
Dzieci w grupie I i w grupie II korzystające z fizjoterapii. Graph 2. Children in group I and group II using physiotherapy.

Należy zwrócić także uwagę na ograniczenia przeprowadzonych badań związane z zastosowaniem MFDR w obszarze diagnostyki wieku chodzenia, obserwowane dziecko może wykonywać zadanie, jednak wykorzystując nieprawidłowe wzorce ruchowe. W związku z tym warto byłoby wykorzystać do oceny motoryki dużej dodatkowe narzędzia, które pozwolą na analizę jakościową prezentowanych przez dziecko aktywności motorycznych.

## Wnioski

1. Wyniki wskazują na istotną statystycznie zależność między poziomem wcześniactwa, a poziomem aktywności prezentowanym przez dziecko w wieku 12 miesięcy od urodzenia.
2. Oceniając poszczególne sfery rozwojowe wśród dzieci urodzonych przedwcześnie, można odnotować mniejszy odsetek dzieci z tej grupy, który podejmuje aktywność zgodną z wiekiem urodzeniowym w wieku 12 miesię-cy w zakresie wymiarów percepcji i samodzielności w porównaniu do dzieci urodzonych o czasie.
3. W grupie dzieci urodzonych przedwcześnie obserwuje się szerszy zakres zmienności w prezentowanym poziomie aktywności, niż w grupie dzieci urodzonych o czasie.
4. Pacjenci mogą prezentować deficyty w poszczególnych obszarach rozwoju w związku z czym proces diagnostyczny powinien być wnikliwy i dotyczyć różnych sfer rozwoju dziecka.
